# Acknowledgment to the Reviewers of *Pharmaceuticals* in 2022

**DOI:** 10.3390/ph16020137

**Published:** 2023-01-17

**Authors:** Pharmaceuticals Editorial Office

**Affiliations:** MDPI AG, St. Alban-Anlage 66, 4052 Basel, Switzerland

High-quality academic publishing is built on rigorous peer review. *Pharmaceuticals* was able to uphold its high standards for published papers due to the outstanding efforts of our reviewers. Thanks to the efforts of our reviewers in 2022, the median time to first decision was 15 days and the median time to publication was 36 days. Regardless of whether the articles they examined were ultimately published, the editors would like to express their appreciation and thank the following reviewers for the time and dedication that they have shown *Pharmaceuticals*:
Aalap ChokshiKuan-Hsing ChenAaron AlfordKuldeep K. BansalAbdalla AwidiKumar GanesanAbdelfattah El MoussaouiKun ZhangAbdelkarim AydiKundi YangAbdelsamed I. ElshamyKun-Eek KilAbdul HafeezKuo-Ching MeiAbdullah Bin ShamsKuo-Shyan LinAbdullah ShaitoKuo-Ting ChenAbhay K. PandeyKwang-Hoon ChunAbhishek ChauhanKyoji MoritaAbhishesh MehataKyoung Sub KimAbraham FinnyKyung-Hyun ChoAbu HazafaKyung-Min KimAbul Kalam AzadKyung-Min LimAdam BuckiKyung-Sun HeoÁdám HaimhofferLacramioara PopaAdam KlimowiczLaia Josa-CulleréAdam Marek PieczonkaLajos GergelyAdina Magdalena MusucLak Shin JeongAdina Turcu-StiolicaLakshmanan GovindanAdolfo Andrade CettoLalitha GummidiAdolfo Gabriele MauroLamiaa M. A. AliAdrian Florin GalLan ZhangAdriana Corina HanganLarisa AnghelAdriana GrigorașLassaâd BelbahriAekkhaluck IntharuksaLasse MarkussenAenim PaeLászló PetriAfang ZhangLászló SzilágyiAfua Adjeiwaa MensahLatifa Rbah-VidalAfzal ShaikLaura Elena SperlingAgata Antosik-RogóżLaura EvangelistaAgata CampisiLaura GattiAgata JanowskaLaura GianottiAgnès HagègeLaura Moreno-MartínezAgnes TelbiszLaura ParmaAgnieszka A. KaczorLaurent DufosséAgnieszka B. OlejniczakLavinia VlaiaAgnieszka GalantyLeandro Augusto CalixtoAgnieszka GrzegorczykLee NguyenAgnieszka Kącka-ZychLeena P. BharathAgnieszka PotęgaLei NieAgnieszka ŚmieszekLei ShiAgnieszka SzewczykLei WangAgnieszka ViapianaLenka StixovaAgnieszka ZagórskaLenuţa ŞutaAgnieszka Zelek-MolikLeonard KhalilovAgnishwar GirigoswamiLeonardo BascoAgustin Rascon-ChuLeonor Puchades-CarrascoAhlem BouhlelLeszek KadzińskiAhmad AinurofiqLetícia Scherer KoesterAhmad G. HegaziLi ZhangAhmad IrfanLia Mara DiţuAhmad JabbarzadehLiana FattoreAhmed A. Abd El-MaksodLianet MonzoteAhmed A. ElhenawyLiang LiuAhmed A. ZakyLiang ZengAhmed Abdal DayemLianliang LiuAhmed Abdel FattahLianne WillemsAhmed E. Abdel MoneimLiat Ashkenazi-HoffnungAhmed ElkamhawyLibin LiuAhmed M. SayedLibor MacurekAhmed RagabLidia Moreira LimaAhmed S. El-ShafieLidia Stasiak-RóżańskaAhmed TemirakLige TongguAhmet Can ErtenLi-Jen LeeAhmet KertmenLilach SoreqAhsan HabibLili FengAilyn FadriquelaLiliana Ostopovici-HalipAishwarya GurumurthyLiliya S. LogoydaAiva PlotnieceLily ChuAjaikumar KunnumakkaraLily Hui-Ching WangAjay BangaLina BaranauskieneAjjampura C. VinayakaLina GaoAjmer Singh GrewalLinnan LiAkhtar RasulLiqing ZangAkihiko NishikimiLirong WangAkira MonjiLisa PilkingtonAklank JainLisa WarnerAkshata NaikLisse Angarita DavilaAlaa Abd-ElsayedLivia CarrascalAlan HarperLivia D’AngeloAlbert PoaterLivia Melo VillarAlberto BaldelliLixin RuiAlberto BartoloméLiyan YangAlberto BongiovanniLiyun YuanAlberto MellaLluvia LópezAlejandro BugarinLoh Teng-Hern TanAlejandro Islas-JácomeLois BalmerAleksandar Ž. KostićLong Chiau MingAleksander BilewiczLoredana Sabina Cornelia ManolescuAleksander BurilovLorenz LeitnerAleksandr S. KazachenkoLorenzo GuazzelliAleksandra GrzelakowskaLorenzo MagrassiAleksandra JózefczykLoretta LazzaratoAleksandra M. BondžićLothar BreckerAleksandra MarciniakLuan Luong ChuAleksandra SzopaLuana Da Silva Magalhães ForeziAleksandra Wisłowska-StanekLubos CipakAleksei MachulkinLuca AmbrosioAleksey D. DrozdovLuca FilippiAleksey Michailovich ChaulinLuca PalazzoloAlessandra AmmazzalorsoLuca Steardo Jr.Alessandra BoschiLuca UnioneAlessandra FerramoscaLucas D. DiasAlessandra QuartaLucas PizzutiAlessandra StacchiottiLucia BaldinoAlessandro IeraciLucia CarboniAlessandro MaugeriLucia CatucciAlessandro ParodiLucia MontenegroAlessio BocediLucia PanzellaAlessio Filippo PeritoreLucia PintilieAlessio GnerucciLucia StančiakováAlessio LodolaLucia TricaricoAlexander DornLuciana ScottiAlexander E. BerezinLucie-Marie ScailteuxAlexander S. NovikovLucinda V. ReisAlexander ShestopalovLúcio FrigoAlexander V. MaltsevLucjusz ZaprutkoAlexandra BarganLucreția UdrescuAlexandra EhrensLudmila KazdovaAlexandra ZakharenkoLudmila Otilia CintezaAlexandru Bogdan CiubaraLuigi Alberto PiniAlexandru GrumezescuLuigi BennardoAlfizah HanafiahLuigi CavannaAlfonso Olaya AbrilLuigi MenghiniAlfredo Téllez-ValenciaLuigi SantacroceAli IrfanLuís Cláudio Nascimento da SilvaAli M. AlqahtaniLuis Cobos-PucAli OzcanLuis Enrique GomezAli ZarrabiLuis Exequiel IbarraAlia TelliLuis G. AlvesAlice S. PereiraLuís Pinto Da SilvaAlice Verticchio VercellinLuise LopesAlicja ChrzanowskaLuiz Carlos SalayAlicja KluczykLuiz Felipe Domingues PasseroAlina BoraLukas D. LandeggerAlina Gabriela RusuLukáš KubalaAlina PǎunescuŁukasz BalewskiAlina Pyka-PająkŁukasz DobrekAlirica SuarezLukasz KozlowskiAlisha PrasadŁukasz ŁopusiewiczAlistair Victor William NunnŁukasz SobottaAl-Khrasani MahmoudŁukasz ŚwiątekAlla MitrofanovaŁukasz SzokaAllen BrooksLuke HunterAlmudena PorrasLuke R. OdellAmalia StefaniuLuminita CrisanAman KarimLuminita MarinAmanda Carroll-PortilloLydia VisserAmandeep SinghLynnette CaryAmany RagabMa. Xenia G. IlaganAmbreen ShoaibMacarena Celina Cáceres LeónAmeeduzzafar ZafarMachireddy Gnaneswara ReddyAmeer Fawad ZahoorMaciej DaniszewskiAmelia CataldiMaciej JarzębskiAmena AliMaciej PrzybyłekAmgad AlbohyMaciej SzaleniecAminu ImamMaciej W. SochaAmir AshoorzadehMadaswamy S. MuthuAmit PandeyMadele DykAmjad HussainMadhulika PradhanAmmar AltemimiMadhumita ChatterjeeAmparo Sánchez NavarroMadjid SoltaniAmr FoudaMafalda R. AlmeidaAna AlcudiaMagda AbdellattifAna BaylinMagda Rhayanny A. FerreiraAna BettencourtMagdalena Chottova DvorakovaAna BorotaMagdalena KotańskaAna Borrego-SánchezMagdalena Krajewska-WłodarczykAna CazacuMagdalena MachowskaAna D. Popović-BijelićMagdalena Makarska-BialokozAna Estela LedesmaMagdalena MalikAna J. Jiménez-AraujoMagdalena MititeluAna M. Carmona-RibeiroMagdalena OsialAna M. SahagunMagdalena Wojciak-KosiorAna Marta De MatosMaged E. MohamedAna MornarMagimairajan Issai VananAna PilipovicMahdy RanjbarAna Rey RiosMahendran SekarAna Rita Xavier De Jesus GameiroMahesh BasavarajuAna TomasMahesh PattabiramanAna TomićMahmoud ElfakyAna V. González-de-PeredoMahmoud ElsayedAna-Maria PutzMahmoud KandeelAna-Maria UdreaMahwash MukhtarAna-Marija DomijanMaida Čohodar HusićAnas M Abdel RahmanMaitree SuttajitAnastasiya SolovievaMaja Jazvinšćak JembrekAnca RoseanuMaja MatulićAnca Roxana PetroviciMajid KomijaniAnca StanaMajid Sharifi RadAnca ZanfirescuMakoto FurugenAnca-Olguța OrzanMakoto HasegawaAnđelka KovačevićMakoto TsunodaAnderson Oliveira SouzaMakram MerimiAndersone AnnaMalček MichalAndleeb KhanMałgorzata GrabarczykAndrás BallaMałgorzata JarończykAndras BikovMałgorzata JeleńAndrás RabMałgorzata Kotula-BalakAndras SzaboMałgorzata KujawskaAndré Barembaye SagnaMałgorzata Maciążek-JurczykAndré FarkouhMałgorzata OstrowskaAndré Moreni LopesMałgorzata SztankeAndré Rolim BabyMałgorzata TabaszewskaAndré SilvaMałgorzata Tyszka-CzocharaAndrea AntosovaMaliheh SafaviAndrea ArsiccioMalik Zaka UllahAndrea BeccioliniMallesh KurakulaAndrea BerenyiovaMamdouh SamyAndrea CiminiMamoona RaufAndrea D. ThompsonMamoru TanakaAndrea DefantMan Kin WongAndrea Herrero-CerveraManel Ben HammoudaAndrea MarkovinovicManisha SharmaAndrea P. RossiManit NuinoonAndrea PiccinManju M. ChandraAndrea RónaváriManoj GargAndrea SpallarossaManoj Kumar TembhreAndreadi AikateriniManuel A. ValdebranAndreas BrachnerManuela CalinAndreas LinkManuela CurcioAndreas ReiberManuela MalatestaAndreas S. KalogirouMara CarsoteAndreas SchmidMarawan AbdelBasetAndreas WeberMarc TiusAndreea IacobMarcela-Elisabeta Barbinta-PatrascuAndrei HonciucMarcella BassettoAndreia FreitasMarcello CasertanoAndrej PerdihMarcello PintiAndrew BurgoyneMarcelo Augusto ChristoffoleteAndrew KatsifisMarcelo Augusto Marretto EsquisattoAndrew T. LucasMarcelo FranchinAndrew ThompsonMarcin OżarowskiAndrey Zamyatnin Jr.Marcin PorebaAndrzej BąkMarcin RóżalskiAndrzej BojarskiMárcio RodriguesAndrzej CendrowskiMarco A. Loza-MejíaAndrzej SlominskiMarco A. Velasco-VelázquezAndy Po-Yi TsaiMarco AllinoviAneliya MilanovaMarco Antonio Loza-MejíaAneta Ostróżka-CieślikMarco Bauzá ThorbrüggeAneta PopovaMarco BiagiAneta ŚcieżyńskaMarco CerranoÁngel AmestyMarco ContardiAngel Josabad Alonso-CastroMarco FornasierAngela BonaccorsoMarco G. SchiavoneAngela MontoneMarco MelladoAngela Patricia HernandezMarco PaolinoAngela SalimMarco PevianiAngela SardoMarco RohdeAngela SorrentinoMarcos Leoni Gazarini DutraAngelika Baranowska-ŁączkowskaMarcos Soto-HernándezAngeliki GiannoulisMarcus Tullius ScottiAngelina AngelovaMarek DrozdzikAngelo SpadaroMarek FoksińskiAnibal De Freitas Santos JuniorMarek KieliszekAniello MaieseMarek WalczakAnil Kumar KalvalaMarek WesołowskiAnil Mathew TharappelMargaret WorrallAnil SinghMargarita Canales-MartinezAnirudh SharmaMargarita D. ApostolovaAnisuz ZamanMargarita E. NeganovaAnita BakraniaMargarita Vega-HolmAnja JoachimMargherita CacaciAnja KolaričMargherita MaioliAnja SaalbachMargherita NeriAnjan MotamarryMaria Addolorata MariggiòAnjaneyulu DirisalaMaría Adelina Jimenez ArellanesAnkit K. RochaniMaria Anele RomeoAnkur KaulMaria Annunziata CarluccioAnkur SoodMaria Antònia BusquetsAnna B. VolnovaMaria AtanassovaAnna BajekMária Budai-SzűcsAnna BaldisserottoMaría C. ArufeAnna BielenicaMaria C. TeixeiraAnna CostagliolaMaria Carmela Bonaccorsi Di PattiAnna DrabczykMaria Conceição OliveiraAnna Duda-MadejMaria da Graça P. M. S. NevesAnna Jamroz-Wis̈niewskaMaría De Lourdes García MagañaAnna JurowskaMaría del Carmen Valls MartínezAnna Karin BelfrageMaria DeRosaAnna Kiełtyka-DadasiewiczMaria DoligalskaAnna KrupaMaria Elizabeth TiritanAnna LesniakMaria Ermelinda S. EusébioAnna M. HawryłMaría F. Tano De La HozAnna Maria CostaMaria Fernandez-HerreraAnna Merecz-SadowskaMaria Grazia SignorelloAnna Merwid-LądMaría I. VaccaroAnna Palko-LabuzMaría J. OrtegaAnna PartykaMaría Jesús HerreroAnna PerriMaria João CebolaAnna Rzepecka-StojkoMaria João RamalhoAnna S. DavydovaMaría José de Jesús ValleAnna SerefkoMaria KoufakiAnna StefanskaMaría Luisa Del Prado AudeloAnna VarizhukMaria M. M. SantosAnna Wiktorowska-OwczarekMaria Manuela GasparAnna WorthmannMaria MarcheseAnnalisa Del PreteMaria NarożnaAnna-Maria PsarraMaria Natalia CalienniAnnarita StringaroMaria Pia DonzelloAnoop KumarMaria RussoAnroop NairMaría Socorro Espuelas MillánAnshul JadliMaria Valeria RaimondiAntero J. AbrunhosaMaría Vivero-LopezAnthony William ColemanMariaevelina AlfieriAnton StaudenherzMariamena ArbitrioAnton V. DolzhenkoMariana CamargoAntonella CasiraghiMariana MatiasAntonella SmeriglioMariana Varna-PannerecAntonia FeolaMarianna CarboneAntonia MancusoMarianna KapetanouAntonina ArgoMarianna RanieriAntonio Di MartinoMariarita BrancaccioAntonio Di StefanoMariarosaria MilosoAntonio Eduardo Miller CrottiMariasole PerroneAntonio Flores-BurgessMaribel NavarroAntonio G. SolimandoMarie BrandtAntonio GidaroMarija-Magdalena PetrinovicAntônio J. RibeiroMarijana KončićAntonio Martins Figueiredo NetoMarilda De Souza GonçalvesAntonio Rodríguez-MorenoMarilia BarrecaAntonio RomanelliMarina Aleksandrovna DarenskayaAntonio RomanoMarina FilimonovaAntonio Rosales MartínezMarina KostićAntonio RussoMarina Zaric KonticAntonio ScaranoMarinela BostanAntónio Sebastião RodriguesMario AbateAntonio VallanoMario CioceAnu AiraksinenMario SimirgiotisAnupam Nath JhaMario Vincenzo RussoApostolos E. PapaloisMariola KozłowskaApostolos ZaravinosMarios G. KrokidisArasu GanesanMarios SpanakisAravinda PaiMarisol López-LópezArcady PutilovMarius ColinArchana MishraMarius Emil RusuAri MeersonMariusz MojzychAriana HuditaMariusz SikoraArif KhanMariusz Tomasz DziadasArijit A. AdhikariMariya PakharukovaAri-Pekka KoivistoMarjolaine GossetArkadiusz P. MatwijczukMark Andrew WhiteArlek González-JamettMark C. HowellArnab MukherjeeMark G. ClemensAroonsri PripremMark J. RobertsonÁrpád TósakiMark T. QuinnArpan AcharyaMark WelkerArpita ChatterjeeMarko JukićArtem D. RogachevMarko KrstićArthur D. TinocoMarko ZdravkovićArtur BeberokMarkus FendtArtur PałaszMarkus MandlArtur RatkiewiczMarlena SzalataArtur TurekMarlena ZyśkArulraj RamalingamMarlene BenchimolArumugam Vijaya AnandMarole M. MalulekaArun UpadhyayMarta CarnovaliArũnas RamanavičiusMarta De AngelisArvind NegiMarta Denel-BobrowskaAshfaq AhmadMarta JarczewskaAshish AgrawalMarta Karaźniewicz-ŁadaAshkan RashadMarta KepinskaAshkan ZandiMarta MenegazziAshok KakkarMarta StrugaAshraf El-BindaryMarta Tsirigotis-ManieckaAshraf M. OmarMartha Patricia Gallegos-ArreolaAshutosh PandeyMartin BrezaAsko UriMartín E. RabassaAsmaa Fahmy KhafagaMartin KuchařAsta JudžentienėMartín Pérez-SantosAstrid KostersMartin PrusinkiewiczAswin L. MenkeMartin SchepelmannAthanasios PapakyriakouMartin TeraaAthanasios TragiannidisMartin VlkAtheer AwadMaruti Jayram DhanavadeAthina GeronikakiMarvin Antonio Soriano-UrsúaAtilla KormosMarwa A. A. FayedAtmika PaudelMarwa MatboliAura RusuMaryam ArdalanAurelia VișaMaryam DaneshpourAurélie SchoubbenMaryam ParhizkarAurélien DeniaudMarzia OgnibeneAvik DuttaMarzia VasarriAwad ShehataMaša Skelin KlemenAwadh YadavMasahiro ShinodaAxel H. SchönthalMasahiro YasunagaAya Naiki-ItoMasahito KatsukiAyat A. AllamMasahito ShimojoAyca Yildiz-PekozMasako NakanishiAyman MahmoudMasashi ToyodaAyoub KasratiMasayuki ItohAyrat ZiganshinMashooq Ahmad BhatAzadeh A. T. BorojeniMasoud RahmatiAzadeh NilghazMasoud Salavati-NiasariAzhar AliMassimiliano EspositoAzizah MalebariMassimiliano RunfolaAzouzi SlimMassimiliano RuscicaBalasubramani ParanthamanMassimo BergerBalasubramanian PalaniappanMassimo ConeseBalazs Istvan TothMassimo TusconiBancha YingngamMateusz KurekBandar A. BabgiMateusz WaliczekBarbara BorczakMathilde Body-MalapelBarbara De FilippisMati KarelsonBarbara MarengoMatias MosqueiraBarbara MiroslawMats EnlundBarbara MognettiMats ErikssonBart De GeestMatteo BiancoBartłomiej GrygorcewiczMatteo Di GiosiaBartłomiej KostMatthew P. HirakawaBartosz PulaMatthias GimpelBartosz TrzaskowskiMauricio Comas-GarciaBeata Sarecka-HujarMauricio MoraisBeata Szulc-MusiołMaurício Temotheo TavaresBeatrix SuessMaurizio NordioBeatriz Godínez-ChaparroMaurizio PaciBeatriz LimaMauro Manuel Martinez PachecoBegoña CerdáMauro MontalbanoBehrooz H. YousefiMax LeongBelén AltavaMaxim PuchkovBen JonesMaxime PichonBenjamin CaroneMay-Ahmed ShawkiBenjamin KochMayank PatelBenjamin PinedaMaytham HusseinBenny RyplidaMayumi NishiBeom-Jin LeeMayur K. LadumorBeow Keat YapMazen NazalBernadette CoricaMazloom ShahBernat SoriaMd Badrul AlamBernhard BiersackMd Golam SharoarBernhard MichalkeMd Jamal UddinBertrand LiagreMd. AdnanBerwin Singh Swami VethaMd. Imtaiyaz HassanBhaskar BiswasMd. MohibbullahBhaswati SenguptaMd. RizwanullahBhupendra PrajapatiMd. Sanower HossainBiagio BaroneMehdi AlikhaniBianca NitzscheMehdi GhasemiBiljana ArsicMehraju LoneBiljana Đ. GlišićMei-Chin LuBimal Kumar GhimireMei-Hsiang LinBin JiangMei-Hwa LeeBingde ZhengMelánia BabincováBing-Huei ChenMelanie R. Power CoombsBin-Nan WuMelgardt De VilliersBiserka RelicMeltem Ezgi DurgunBishoy Y. A. El-AaragMengxi ShenBishweshwar PantMengyang FengBissera PilichevaMepur RavindranathBiswajit PadhyMeritxell García-QuintanillaBiswajit PandaMh Busra FauziBlanca Elí Ocampo-GarcíaMi Young LeeBn Vedha HariMichael DanilenkoBo SunMichael HöpfnerBo Young ChungMichael John PlaterBoban AndjelkovicMichael SwansonBogdan Ionel TambaMichael Van DykeBogdan SevastreMichał BurdukiewiczBoleslaw T. KarwowskiMichal MajewskiBono LučićMichał OtrębaBoohwi HongMichal ŘezankaBorislav AngelovMichal TomczykBorislav KovačevićMichel MarreBorja SaezMichel PucéatBoryana Nikolova-MladenovaMichel YeglesBorys PrzemyslawMichele CiullaBratko FilipicMichele Di CosolaBrenda LeeMichele GraciottiBrent A. BakerMichele LaiBrent SieskyMichele RussoBrian MonkMichelle Lima GarcezBrice WilsonMiguel Gomes GuerraBrigitte MarianMiguel HuertaBrijeshkumar PatelMiguel OrtegaBrînduşa Alina PetreMihai Cosmin CenariuBrindusa TiperciucMihai MusteataBruno FigadereMihail BirsaBruno TassoMihnea ZdrengheaBuddhadev LayekMijung KwonBuǧra KarasuMikael JensenBurkhard BrandtMikhail M. KostikByung Seok MoonMikio HayashiC.Benjamin NamanMikko Uusi-OukariCălin Gabriel FloareMilan ČížCamelia TulcanMilan SencanskiCamila Squarzoni DaleMilena Magalhães AleluiaCamillo RosanoMilena PetkovicCandace R. LewisMilica AćimovićCao-An VuMilica BorovcaninCarla FernandesMilica MarkovicCarla FiorentiniMilica PopovicCarla LopesMiloš I. DjuranCarla Roberta De Oliveira CarvalhoMilton KanashiroCarla RozzoMina LeeCarlene MooreMing HuangCarlo AprileMing ZhangCarlo GalliMingfu ChenCarlo MusioMing-Jay DengCarlos A. García-GonzálezMingyou HuCarlos GeraldesMinwei ZhangCarlos Gutiérrez-MerinoMinyi TianCarlos H. HirokiMiran SebestjenCarlos Portugal-NunesMircea Ioan PopaCarlos RosadoMircea TampaCarlos Torres-TorresMirella GiovarelliCarlotta PontremoliMiriam HickeyCarmel StockMirjana B. ČolovićCarmen DinizMirkka P. SarparantaCarmen GianfraniMiroslav NovakovićCarmen JeronimoMiroslava KačániováCarmen Lizette Del Toro SánchezMirosław AniołCarmen Méndez HernándezMiroslawa PuskulluogluCarmen PanaitescuMititelu Tartau LilianaCarol LinMitsuru NaitoCarolina DaviesMitsutoshi SetouCaroline Costa MoraesMladena Lalic-PopovicCaroline L. NgMobashir MohammadCarsten CulmseeMohamad YasminCarsten ZeilingerMohamed A. A. OrabiCassandra FlemingMohamed A. AbdelgawadCassiano Felippe Gonçalves de AlbuquerqueMohamed AbdelgiedCaterina De LucaMohamed Abdullah JaberCaterina FedeMohamed El RaeyCaterina Maria GambinoMohamed El-MeseryCaterina PalleriaMohamed FodaCatherine TuleuMohamed OrabiCatia LambertucciMohamed Osman RadwanCecilia Zavala-TecuapetlaMohammad Asif SherwaniCélia CarliniMohammad BasyuniCelina WojciechowskaMohammad Hojjat-FarsangiCennet OzayMohammad Imran KhanCerasela Elena GîrdMohammad QneibiChahrazade El-AmriMohammad Reza MozafariChai-Lin KaoMohammad ShariareChan Young ShinMohammad Tavakkoli YarakiChander Parkash DoraMohammed BuleChandra PrakashMohammed H. MohammedChandra Sourabh AzadMohd AdnanChandrabose SelvarajMohd AsgherChandrashekhar VoshavarMohd Ezuan Bin KhayatChang Yeon YuMohd ImranChangiz TaghibiglouMohd Nazam AnsariChan-Yen KuoMohini GuleriaChao LeiMohit BibraChao ZhouMoisés Tolentino B. Da SilvaChao-Lin LiuMojtaba MokariCharles L. WilkinsMojtaba VaismoradiCharles NortonMominur RahmanCharles ShulerMona Abdel-TawabCharles TruilletMona BatishCharles W. SchindlerMona M. El-RefaeyCharupong SaengboonmeeMonica GulatiChellappagounder ThangavelMonica Lisa Fernandez-QuinteroChen Huei LeoMónica Ríos-SilvaCheng ZhuMonica Y. LeeChengwen SunMonika Kadela-TomanekChen-Hung LeeMonika Les̈kiewiczChennaiah AndeMonika PapieżChenyu SunMonika PrzeorCheorl-Ho KimMonika ScheerChia-Chun TsengMonirul IslamChian Ju JongMontserrat GarcíaChiara BrulloMonwadee WonglapsuwanChiara CaselliMoon Il KimChiara Da PieveMorteza KafshgariChiara MacchiMostafa A. HussienChiara Paola ZoiaMotohiro OkadaChiara PavanelloMourad A. M. Aboul-SoudChien-An ChenMozaniel Santana de OliveiraChi-Hao TsaiMrinmoy GhoshChih-Hua TsengMrutyunjay JenaChih-Peng LinMudassir Hussain TahirChihping HsuMuhamad MustafaChin-Chuan HungMuhammad Bilal SadiqChing-Chung HsiaoMuhammad Imran RahimChingfeng WengMuhammad IqhrammullahChinh Tran To SuMuhammad JunaidChinna BathulaMuhammad Khalid KhanChirag N. PatelMuhammad NawazChisato KinoshitaMuhammad RasoolChi-Ting HorngMuhammad Sajid HussainCho Yeow KohMuhammad Shahid IqbalChoon Fu GohMuhammad Usman MunirChris LabakiMuhammad YasirChristel VangestelMuhammad ZubairChristian BarbatoMuhittin KulakChristian BleilevensMumtaz AnwarChristian Erick PalavecinoMunawar NaylaChristian GorzelannyMuralikrishnan DhanasekaranChristiana M. NeophytouMurat AladaǧChristiane Fernandes HornMurat BingulChristin NeuberMurugesan ChandrasekaranChristina Barja-FidalgoMusaddique HussainChristoph HugenschmidtMustafa SevindikChristophe MalletMustafa TurkyilmazogluChristophe PadoinMustapha Cherkaoui-MalkiChristopher AsquithMuthu SathishChristopher CoteMuthuramalingam PandiyanChristopher DoeringMutien-Marie GariglianyChristopher PlaisierMuyun XuChrong-Reen WangMuzamil KhatriChuan ChenMyron R.. SzewczukChuen-Fu LinMyunggi YiChun GuoN.Jeelan BashaChun HuNadezhda M. BazhanChun Nam LokNadia Elghobashi-MeinhardtChun PanNadine BernhardtChun WangNadine JagerovicChun ZhangNadja FreundChungchun WuNagah ArafatChung-Ming ChenNagaraju KerruChun-Han ChenNagavendra KommineniChun-Liang ChenNagendra ChauhanClare HoskinsNagesh KolishettiClarence T. T. WongNajla AltwaijryClaudia Avitia-DomínguezNallely Rivero-PerezClaudia Di GiacomoNam Sook KangClaudia Duran-AniotzNan HaoClaudia DurantiNancy GavertClaudia PennaNanlin WuClaudia PisanuNaoki YamamotoClaudio FerranteNapoleão Martins Argôlo NetoClaudio RossiNarendar DudhipalaClaudiu MorgovanNarendra Pal Singh ChauhanClaudiu N. LunguNassim Ghaffari-Tabrizi-WizsyClemens KratochwilNatali HudzClemens ZwergelNatalia BelosludtsevaCody CrosbyNatalia KrastevaCody PeerNatalia Mayumi InadaColin C. SeatonNatalia SizochenkoConcetta AltamuraNatalija PolovicConcettina La MottaNatasa Bogavac-StanojevicCongbao KangNatércia ConceiçãoConstanze HilgendorfNavadeep ShrivastavaConxita MestresNavaz KharazianCorreia Joana SofiaNaveen RaghavendraCosima Damiana CalvanoNawaf Al-MaharikCosmin Mihai VesaNayely Leyva-LópezCrina MuresanNazar TrotskoCristian MornoşNeamtu BogdanCristian P. LunguNeda S. GavarićCristian RipoliNeelesh Kumar MehraCristian ScheauNeeraj SoniCristiano CarbonelliNeetu DayalCristiano J. De AndradeNeli Kinga OlahCristina GhiciucNelson FerreiraCristina MaccalliniNemany HanafyCristina Martin-SabrosoNenad JoksimovićCristina MinnelliNermin EissaCristina MüllerNeslihan Üstündaǧ OkurCristina OlivaroNeveen SaidCristina SatrianoNeven PapicCristina SobacchiNibedita LenkaČrtomir PodlipnikNic M. GillingsCsaba HetényiNicola MicaleCvijeta Jakobusic BralaNicola PuglieseCyril BařinkaNicola SalvareseCyril FersingNicola SgherzaCyrus MotamedNicolae BacalbaşaDae Hwan ShinNicolas LepareurDaeui ParkNicole NaumannDag Erlend OlbergNicoleta Nicole RaduDahong LiNicoletta PedemonteDaisuke MiyamotoNidal JaradatDaisy VolmerNiels Damkjær OlesenDalal Hammoudi HalatNikesh GuptaDâmaris SilveiraNikola MarakovićDamian BartuziNikolaos AndreatosDamião Pergentino de SousaNikolaos NazirisDana NiculaeNikolaos StalikasDaniel A. AbugriNikolay PolyakovDaniel ArcanjoNikolay V. GoncharovDaniel BaeckerNikole ByrneDaniel Carneiro MoreiraNikolett Kállai-SzabóDaniel Florin LighezanNil PandeyDániel NemesNilesh ChoudharyDaniel P. OttoNing QuDaniel RoethNirupama RamadasDaniel SzulczykNisar Ul KhaliqDaniela ArosioNitin CharbeDaniela C. PaseroNitin GuptaDaniela CarboneNitin KhandelwalDaniela Carmen AbabeiNives GalićDaniela Elena PopaNoam ZevitDaniela HanganuNobuaki YasuoDaniela ImperioNobuhiro KanemuraDaniela MiricescuNobuhiro OobaDaniela ValentiNoelia DuarteDaniele BaniNoelle S. WilliamsDaniele CavaleriNoemí RotllanDaniele LecisNoha F. AbdelkaderDaniele NovielloNoppawan MoralesDaniele VergaraNorberto P. LopesDaniil N. BratashovNorhana JusohDaocheng WuNorio SogawaDaozong XiaNorma G. Padilla-EguiluzDario Di SilvestreNorrie PearceDariusz Maciej PisklakNoureddine IssaouiDariusz T. MlynarczykNovriyandi HanifDarya NovopashinaNuno ValeDashevskiy AndriyNunzio DenoraDavid B. AlexanderNupur DasDavid BalayssacNuray AcarDavid ChatenetNurhasni HasanDavid Chávez-FloresNury Pérez-HernándezDavid DixonOana Andreea CojocaruDavid LeitschOana Cristina ŞeremetDavid M. PereiraObinna ObianomDavid SinkaOctavian Tudorel OlaruDávid SzatmáriOihana GordobilDavid ZweikerOkezie EmmanuelDavide MaggiOksana SytarDavide MoiOleg A. RakitinDavor PlavecOleg DenisenkoDébora Do Rocio KlisiowiczOleg Ivanov KolodiazhnyiDebottam SinhaOleg ShuvalovDeepak K. LokwaniOlga A. GoryachevaDeepak KulkarniOlga AfanasievaDeepak KumarOlga Di FedeDeepak Kumar KhajuriaOlga KopachDelia Ioana HorhatOlga LuzinaDelia MezzanzanicaOlga SaikDelphine MikaOlga YarovayaDenis BuxtonOlga Yu BakulinaDenis E. KainovOlha MykhailenkoDenis S. KudryavtsevOlimpio MonteroDenis V. VoroninOlivier DeschaumeDenisa MarginǎOlivier SorgDennis BrownOlja Lj ŠovljanskiDennis FlanaganOluwatoyin A. AdelekeDennis V. CokkinosOmar El HibaDerek J. B. KleinveldOmar NomanDevaraj BharathiOmkara Swami MuddinetiDevi Prasan OjhaOndřej SlanařDevis BenfaremoOrawan WongmekiatDhananjay YadavOrnella GuardamagnaDhanya GeorgeOsama A. ElkashtyDharmendra ChoudharyOscar CasisDharmeshkumar PatelÓscar Lorenzo GonzálezDiana Andreia Tavares PintoOtavio ThiemannDiana AntalOtilia BobisDiana Camelia NuţǎOuldouz GhashghaeiDiana CholakovaOvidiu OnigaDiana Cláudia PintoOxana DobrovinskayaDianbao ZhangOzgul GokDidier DesmaëleOzioma NwaborDidier DevaursPál HerczeghDillon MintoffPalaniyandi KaruppaiyaDima SabbahPandurangan RamarajDimitra ChatzileontiadouPankaj SharmaDimitri GilisPanoraia I. SiafakaDimitrios KouretasPaola BarrajaDimitris C. ChatzopoulosPaola CartaDimosthenis StamopoulosPaola DonatoDina El-KershPaola GavazzoDinithi PeirisPaola GratteriDiogo Rodrigo Magalhaes MoreiraPaola PiomboniDipankar RoyPaolo BlasiDirk C. AlbachPaolo FagoneDjenisa RochaPaolo MarianiDmitri KosenkovPaolo MazzeoDmitriy BerilloPaolo MonardoDmitriy IvashchenkoPaolo TrucilloDmitry A. StetsenkoPapp Lajos AttilaDmitry KarpovParaskev T. NedialkovDmitry PelageevParaskevas D. TzanavarasDmitry ShulgaParisa GazeraniDmitry ZorovParisa ZiaratiDobrinas SimonaPartha Sarathi AddyDolores R. SerranoParvesh SinghDomenica Donatella Li PumaPascal PineauDomenico AlbanoPatrice Nsangou MimcheDomenico TricaricoPatricia Álvarez-FitzDominic WellsPatrícia BatistaDominik DłuskiPatricia SeverinoDominik KoszelewskiPatrick SchwarzDominique ManikowskiPatrick WeydtDonald TomaliaPatrizia CancemiDonatella Delle CavePatrizia FormosoDong ChenPatrizia Nadia HaniehDong Hee NaPatrizia Polverino de LauretoDong Hyun KimPatrycja KleczkowskaDongdong CaoPaul JoyceDong-Hun BaePaula AntonoaeaDongqing WeiPaula M. T. FerreiraDongWoon HanPaula PintoDong-Yeon KimPaulina KoczurkiewiczDongying LiPaulo GasparDoriano LambaPaulo Jorge PalmaDorota KopciuchPaulo MatafomeDorota Wójcik-PastuszkaPaulo SantosDorota WrześniokPavan Kumar DhanyamrajuDorota ŻelaszczykPavel KomarovDorothee WeihrauchPavel LobachevskyDouglas SteinkePavel SolopovDragos HorvathPaweł KowalczykDragos Paul MihaiPaweł KrukowDrew NassalPawel KrysinskiDuarte MarquesPaweł LisieckiDuc-Thang VoPaweł MierzejewskiDunja ŠamecPawel OlczykDušan DimićPaweł RamosDušan RačkoPaweł SzymańkiE. Maruthi PrasadPayal GangulyEbuka E. DavidPayam ZahediEdgar Paredes-GameroPayel ChatterjeeEdina PandurPedro Aguilar-ZarateEdio MaldonadoPedro AmorósEdit CsapóPedro BullonEdmond GravelPedro Ferreira-SantosEdoardo Marco NapoliPedro MarquesEduardo Collantes EstévezPei-Ling HsiehEduardo González-ZamoraPekka A. PostilaEduardo Gutiérrez-AbejónPeng ZhangEduardo Osiris Madrigal SantillánPeng ZouEduardo Padilla CamberosPer Jr GreisenEduardo ValarezoPer MalmbergEdvardas DanilaPeta HarveyEdward KrzyżakPetar OzretićEdyta MakuchPetar TodorovEgle SolitoPeter C. GainesEgor VerbitskiyPeter D. WestenskowEiichi KumamotoPeter J. ChoiEike Roman HrinciusPeter John JervisEkaterina D. ObluchinskayaPeter KubiszEkaterina GeorgievaPeter ParsonsElena AguileraPēteris TretjakovsElena CicheroPetr ZamostnyElena Del FaveroPetra HeffeterElena DintePetra SchlingElena I. RyabchikovaPetre IonitaElena K. BeloglazkinaPhilip J. KuehlElena LeshchenkoPhilip LangeElena PiniPhilipp J. PoxleitnerElena StoleruPhilipp Paul LobmaierElena UspenskayaPhilipp S. OrekhovElena V. KudryashovaPhilippe BuletElena V. ProskurninaPhilippe De DeurwaerdèreElena VaroniPier Paolo PiccalugaElena ZavyalovaPierpaolo ScaranoEleonora AlfinitoPierre De RossiEleonora VatamanPierre-Antoine BonnetEleonora VirgilioPietro CrispinoElham AhmadianPietro ScicchitanoElia BariPilar HoyosElia M. GruesoPing-Shan LaiEliana Hiromi AkaminePin-Kuang LaiEliana LeoPiotr AlsterEliane de Oliveira SilvaPiotr ChmielarzElias ChristoforidesPiotr DobrowolskiElikaee SamiraPiotr KurcokElina Vuorimaa-LaukkanenPiotr MichelElisa BelluzziPiotr MorasiewiczElisa Lopez-DoladoPiotr OkińczycElisabetta AntonelliPiotr ŚwiątekElisabetta BarresiPiotr SzwedaElisabetta BraviPiroska Szabó-RévészElisabetta CaiazzoPiyush GuptaElisavet GrouziPiyusha P. PagareElisavet StavropoulouPonnulakshmi RajagopalElizabeth Carvajal-MillanPrabhanjan GiramElizabeth HicksPrachi UmbarkarElizabeth S. FernandesPradeep KumarElizaveta KlimanovaPradeep Kumar BollaElizaveta StarodubovaPradeepkiran Jangampalli AdiElke ButtPrakash ThapaEl-Saadony MohamedPramit ChowdhuryEl-Sayed R. El-SayedPranabananda DuttaElvire Pons-TostivintPranee SrirajElwira ChrobakPrasanna Kumar SanthekadurElwira SieniawskaPrasanna RamaniElzbieta JandaPrasannavenkatesh DuraiElżbieta Łodyga-ChruścińskaPrasanth Saraswati AkellaElżbieta Studzińska-SrokaPrashant RajbhandariElżbieta WyskaPrashant TaraleEmad H. M. HassaneinPrathap SomuEman M. OthmanPratibha PandeyEman MazyedPraveen KumarEmanuel Loeza AlcocerPraveen Nekkar RaoEmanuel RaschiPrawej AnsariEmanuele RizzoPrema Latha MallipeddiEmil BulatovPriya BharateEmil PaluchPriya Darshni ShanmugarajahEmilia Janiszewska-TurakPriyanka SinghEmma Adriana OzonPrzemysław DorożyńskiEmmanuel LetsyoPrzemysław DudaEmoke PallPrzemyslaw SiejakEmyr BenbowPumtiwitt C. McCarthyEnade Perdana IstyastonoPushpa Raj JoshiEng Shi OngPutra SantosoEngy A. MahrousQi ChenEngy ElekhnawyQianwei ChenEnrico GugliandoloQilei ChenEnrico RadaelliQingming HuangEnrico RagniQing-Rong LiuEnrique Domínguez-ÁlvarezQingyu ZhouEnzo ScifoQi-Yin ChenEri S. SrivatsanQonita Kurnia AnjaniEric J. WoolfQuintas-Granados Laura ItzelEric MasRabiul IslamErick Paul Gutiérrez-GrijalvaRachid SkoutaErika A. EksiogluRadek PohlErika A. TaylorRadim VrzalErika Azorín-VegaRadomir JasińskiErika CioneRadosław KujawskiErika StaudacherRadu C. RacovitaErmal IsmalajRadu TamaianErwin DreesenRafael De La TorreErzsébet MernyákRafał LoskaEssa SaiedRafał OgórekEstefanía MorenoRafał WienchEstelle LeclercRaffaele CapassoEsther MorenoRaffaele RiccioÉtienne ChatelutRaffaella PetruzzelliEugene Haydn WaltersRahul KavtheEugene KimRahul ShingareEugenia BezirtzoglouRahul Y. MahidaEugenio ProvenzanoRai Campos SilvaEurico LimaRaj KumarEva Češ̈kováRajakrishnan VeluthakalEva MontanéRajarshi GhoshEva PinhoRajendra Prasad SettemEva Siles RivasRajendran Jagathesh Chandra BoseEvangelos LiberopoulosRajesh P. ShastryEvelina Maria GosavRajiv DahiyaEvelyn LamyRakesh K. SindhuEwa Daniela RaczyńskaRakesh TiwariEwa Gibuła-TarłowskaRaluca IanchisEwa OledzkaRama KadambEwa TomaszewskaRamachandran VinayagamEwan PlantRamendra Pati PandeyEwelina HallmannRamesh K. GanjuEwelina KrólRamesh KumarEwelina PatyraRamon GarcÃ­a-DomenechFabiana Andréa MouraRania A. H. IshakFabiana Volpe-ZanuttoRania AlaaeldinFabiano RodembuschRania M. HathoutFabien CailléRanjit DeFábio Filipe Fereira GarrudoRaphaël ThuillierFabio MammanoRaquel AlvesFabio PastorinoRaquel Fernández GarcíaFabio ZobiRathapon AsasutjaritFabrice DarcheRatko LasicaFabrice FleuryRaul G. EnriquezFabrizio CartaRazieh RazaviFacundo MatteaReggie Hui-Chao LeeFacundo RuizRegina KasperekFadime Aydin KoseRéjean CoutureFahad A. AlhumaydhiRemco J. MolenaarFahad KhanRémi RosièreFahad M. AlmutairiRenata Heisler NevesFaiyaz ShakeelRenata MikstackaFani LadomenouRenáta MinoricsFanwang MengRenata SoutoFarid SroorRenata Vidor ContriFarman Ali KhanRenato B. PereiraFarzad PakdelRene BuchetFarzaneh SabbaghRené CsukFatemeh BahadoriRenyer CostaFatemeh TabatabaieReuben Jih-Ru HwuFathi MoussaReza RanjbaranFatima BentoRezvan JamaledinFatima RizviRiaz UllahFátima RoqueRicardo MarquesFatme AlAnoutiRiccardo Di CoratoFayyaz Ur Rehman MuhammadRichard D’ArcyFederica BrunoRichard FunkFederica ChiapporiRichard J. StarkFederica Di BerardinoRichard SheardyFederica FogacciRima HajjoFederica PianiRita Cristina OliveiraFederico CesanoRita KissFederico SireciRita Pei Yeh ChenFedora GrandeRizwan WahabFei XingRob T. StrikerFei YeRobert AncuceanuFelipe A. La PortaRobert KissFelipe Javier Chaves-MartinezRobert MusiołFelipe KazmirczakRobert T. MeansFelix DitzingerRobert W. RobeyFelix Javier Jiménez JiménezRoberta IbbaFelix SternbergRoberta MoschiniFenghai GuoRoberta SalaroliFengjui ChenRoberto ChimenzFeng-Lin YenRoberto CiampagliaFerdinando FiorinoRoberto FrauFerdinando NicolettiRoberto PaganelliFerenc BoldizsárRoberto RoncaFerenc ErdődiRobin L. CooperFermin Sanchez-GuijoRodica OlarFernanda Viana CabralRodica-Mihaela DinicǎFernanda VilarinhoRodman E. TurpinFernanda WirthRodrigo RazoFernando GilsanzRogelio Gónzalez-GónzalezFernando Torres AndónRoger E. ThomasFilip ŠeniglRohan RejFilipe ElvasRohitash JamwalFilipe PenteadoRoksana MarkiewiczFilippo FratiniRoldán-Roldán GabrielFilippo MiglioriniRomain DuvalFirdos Alam KhanRomain EychenneFirzan NainuRoman B. LesykFlavia FerrantelliRoman BegunovFlavia VaranoRomana VulturarFlavia ZacconiRomána ZelkóFlora N. BalievaRomeo RomagnoliFlorencio Jr ArceRonald TaylorFlorentina PluteanuRonaldo Nascimento De OliveiraFloriana VolpicelliRonan MurphyFlorin BorcanRong XuFNU SurakshaRong-Fu ChenForoud ShahbaziRongguo RenFotis BaltoumasRonit SionovFotis IliopoulosRosa CalvelloFranc VrečerRosa Di LiddoFranca CastiglioneRosa María Giráldez-PérezFrancesc Xavier AvilésRosa Maria Guillen FretesFrancesca AielloRosa TundisFrancesca ButtiniRosana Simón-VázquezFrancesca ManciantiRosario González-MuñizFrancesca MusumeciRoshan KatekarFrancesca ReggianiRoshan KumarFrancesco AgostiniRoxana Maria WasnickFrancesco BellinatoRoxana-Maria AmărandiFrancesco CacciolaRoy BeranFrancesco CiodaroRubem F. S. Menna-BarretoFrancesco FerraraRubén Darío Castro-TorresFrancesco FornaiRubén Francisco González-LaredoFrancesco PiacenteRubén Martín-EscolanoFrancesco StellatoRuei-Nian LiFrancisco AlvesRute G. MatosFrancisco Cortés-BenítezRuth PrasslFrancisco Cruz-SosaRuth ThompsonFrancisco Fueyo GonzálezRuyin LiuFrancisco Javier Alvarez-MartinezRyan LimbockerFrancisco Javier Otero-EspinarSaad ShaabanFrancisco Javier Ruiz-OjedaSaadet InanFrancisco José González-MineroSabata PiernoFrancisco LeonSabina AntoniuFrancisco LesSabina PodlewskaFrancisco MendezSabrina L. McIlwrathFrancisco RequenaSabyasachi MoulikFrank BraunSachio TakenoFrank StahlSaeid KargozarFranz-Josef Meyer-AlmesSagrario Martin-AragonFrédéric EbsteinSai Reddy DodaFrédéric VillegaSaikat MitraFredyc DíazSajal SenFritz BoegeSaket PatelFumitoshi HirayamaSakineh Kazemi NoureiniGabriel Del Río GuerraSally Yunsun KimGabriel HancuSalvatore BaldinoGabriel Lucian RaduSamanta RaboniGabriel Navarrete-VazquezSameer JoshiGabriela Castañeda‐CorralSamet ÖzdemirGabriela V. VochitaSamir BarghoutGabriele CarulloSamir ChtitaGabriele CaviglioliSamir Frontino de Almeida CavalcanteGaetano MarvertiSamira MalekmohammadiGaetano SantulliSamuel K. KwofieGagan DhawanSanda AndreiGajdoš Kljusurić JasenkaSandeep Kumar Reddy AdenaGalina LikhatskayaSandip S. ShindeGanesh NikaljeSandor BenyheGang WeiSándor Kunsági-MátéGang YeSandra DonniniGangarapu KiranSandra I. D. SimõesGary GellermanSandra N. PintoGary O. RankinSandra Oramas-RoyoGary StephensSandrine CojeanGatikrushna SinghSandro GlumacGautam SethiSangeeta TiwariGeert Jan SterkSangram SamalGema Mondejar-ParreñoSanja TomićGemma Caterina Maria RossiSanjairaj VijayavenkataramanGengyang YuanSanjay KumarGeorg PetroianuSanjaya Kumar SahuGeorge LambrinidisSanket MishraGeorge Mihai NitulescuSankha BhattacharyaGeorge PavlidisSanoj RejinoldGeorge TsogkasSanta CirmiGeorge VarvounisSantiago Diaz-OltraGeorgia N. ValsamiSantosh BashyalGeorgia-Eirini DeligiannidouSantosh Kumar SinghGeorgios GermanidisSantosh PeddiGeorgios K. MarkantesSaptarshi RoyGeorgios KotzalidisSara FedericiGeorgios TzikosSara Luz Morales-LázaroGerard DumancasSara M. RobledoGerardo CorzoSara PedronGerasimos RassiasSara PerteghellaGergely FehérSara Sáez-OrvizGerhild EulerSara SalucciGerman Dominguez-VíasSara Tariq A. Al-RashoudGermano CastelliSara TombelliGernot ZisselSarah MazzottaGerry LushingtonSarah ShigdarGerson J. RodriguesSarath VijayakumarGesmi MilcovichŠárka ŠtěpánkováGeun Shik LeeSarkar M. Abe KawsarGhadha Ibrahim FouadSascha R. A. AllesGhasem SargaziSasha R. AzarGheorghe IliaSashi DebnathGholamreza DaryaborSathish DyawanapellyGiacomo Dal BelloSathish Sundar Dhilip KumarGiacomo LuciSatomi NiwayamaGiada AmodeoSaurabh MishraGiada CrescioliSaurav Kumar JhaGiampietro RavagnanSaverio CandidoGiancarlo MorelliSawsan A. ZaitoneGianfranco FaviSe kyoo JeongGianfranco PintusSeamus O’ReillyGianluca CarnevaleSebastián BonarddGiau VoSebastian SchloerGil FraquezaSebastian ZahnreichGill DiamondSebastiano IntagliataGiok KimSébastien LacroixGiorgio GiorgiSelvakumar EdwardrajaGiorgio TregliaSeok Kyeong OhGiovanna BaronSeon-Cheol ParkGiovanna BorrielloSerge NatafGiovanni GraziosoSergey BaykovGiovanni LaviolaSergey DikalovGiovanni MatareseSergey FilippovGiovanni N. RovielloSergey SamsonovGiovanni RaugeiSergey UspenskiiGiovanni RibaudoSergio A. RodríguezGiovanni StelitanoSergio BorghiGiovanni TossettaSérgio M. MarquesGiulia GentileSergio MartiGiuliano CiarimboliSerguei KozlovGiuliano SiligardiSerhat Sezai ÇiçekGiulio CeolottoSerhii LupenkoGiulio FracassoSeung Hyun KimGiulio VistoliSeung-bae YoonGiuseppe Antonio MalfaSeyed Alireza R. SalamiGiuseppe CirilloSeyun KimGiuseppe LanzaShadeed GadGiuseppe ZanottiShah KhanGlaecir Roseni Mundstock DiasShahin HomaeigoharGlaucio Valdameri ValdameriShahla MirzaeeiGleiston DiasShailendra S. GuravGloria ManzottiShams TabrezGloria María Molina SalinasShangang ZhaoGobinath ShanmugamShankar Jaikishan EvaniGolara GolbaghiShanshan TanGopichand GuttiShao LiGopinadhanpillai GopakumarShaobo RuanGopinath VenkatramanShao-Hsuan KaoGoran GajskiShaoli LinGoran ŠinkoShaoquan ZhengGouri S. JasShaoxun WangGovindasamy ManiShariful IslamGowthamarajan KuppusamyShashank KumarGraça SoveralShaza Al-MassaraniGraeme BolgerShekhar NeemaGregor KravanjaShekhar SahaGregorio PeronShengbao CaiGregory CaputoSherry D. FlemingGrigoris ZoidisShiang-Jong TzengGrzegorz CholewinskiShibei WuGrzegorz CieslarShichao BiGrzegorz HelbigShigehiko OgohGrzegorz SchroederShigeki SetoGrzegorz TrybekShigenori TanakaGuangde JiangShigeo OhtaGuanglei WuShigeto TakebayashiGuang-Zhi ShanShijie HeGuan-Jhong HuangShin MatsuokaGuendalina ZuccariShindu ThomasGuglielmo VeronaShinichi SakamotoGuiliang ChenShining LooGuillermin Agüero-ChapinShizhao LiGuillermo Estivill-TorrúsShogo HamaguchiGuliz ArmaganShogo MoriGunars DubursShogo OzawaGunnar AntoniShoib Sarwar SiddiquiGuodong ShenShoji OuraGuo-Fu LiShoko ItakuraGuohua MaShraddha ParateGuoqing NiuShuang LiuGuoyu PanShuang SongGurpreet Kaur AroraShu-Ling TzengGustavo D. ParisiShun DongGustavo Duarte PimentelShun-Fa YangGustavo PimentelShyi-Long LeeGuus A. M. S. Van DongenShymaa EnanyGwénolé LoasSibel İlbasmiş TamerGyorgy KeseruSiddhartha PatiGyörgy Tibor BaloghSiew-Keah LeeH. N. Aswatha RamSijin GuoHaigang WuSilvia AlbertHaim GrebelSilvia BordoniHaisu ZengSilvia De FranciaHaiyan TanSilvia GiovanniniHaiyang LiuSilvia LandiHalima KerdjoudjSilvia LocarnoHamid IrannejadSilvia MironeasaHamid RashediSilvia PasquiniHamza MechchateSilvia VasiliuHanan HanaSimon LucaHang YangSimona CandianiHangang YuSimona CitroHani Nasser AbdelhamidSimona ClichiciHan-Joo MaengSimona De VitaHanna BandarenkaSimona FellettiHanna SzaeferSimona Funar-TimofeiHannes TodtSimona MoldovanuHans De LoofSimone DonatiHany Hamdy ArabSiti Rafidah YusofHao ChenSiu Wai ChoiHao-Jen HsuSiva PandaHarbinder SinghSivashanmugam AmirthalingamHardeep Singh TuliSlavica KvolikHarris PratsinisSławomir JakiełaHaruhisa KikuchiSławomir MilewskiHarvey G. RowethSobhi M. GomhaHarvey HouSofia MavrikouHassan AlbarqiSofia MitakouHassan Al-KaragolySofia VasilakakiHassan AlmoazenSohail Anjum ShahzadHassan GhonaimSoheb Anwar MohammedHassan Y. EbrahimSok Kuan WongHassan ZafarSolmaz Maleki DizajHayder HeyamSonam MittalHayrettin O GulcanSónia CarabineiroHeba A. S. El-NasharSonia De CastroHeba SahyonSonia Escandón-RiveraHedvig BölcskeISonia LaneriHee Eun KangSonia PinhoHee Kyoung JooSoo-Hong LeeHee-Chul AhnSophia S. BorisevichHeike WeighardtSophie TamareilleHeiko HeerklotzSophie Thetiot-LaurentHeiko MöllerSotiris HadjikakouHeinz GrunzeSowjanya ThatikondaHelder MarcalSpyridon PapageorgiouHelena Bustos-CruzSrđan BanacHerbert Ryan MariniSri MeliaHesham El-SeediSrihari KonduriHester DoyleSrikanth GattuHiba NatshehSrinath PashikantiHideaki FujiiSrinidhi JayaramanHidehiko KikuchiSrinivas AjjarapuHidehiro UekusaStane SrčičHidekazu KawashimaStanislav KotlyarovHideki AiharaStanisław KaliszHideo KatoStefan Cristian VesaHideo WadaStefania ButiniHideyuki SatoStefania CerutiHie-Won HannStefania ContiHimanshu JenaStefania MerighiHirofumi HanaokaStefano BellostaHirokazu SakamotoStefano De BenedettiHiromichi YumotoStefano DugheraHironobu AsaoStefano RealeHiroshi SakaueStefanos LeontopoulosHiroshi WakabayashiStéphane BurteyHiroyuki AonoStéphanie BriançonHiroyuki FukuiStephanie DuguezHiroyuki KimuraStephen LewisHiroyuki TsuchiyaStephen T. AbedonHiroyuki WakiguchiStergios PispasHisayuki KomakiSteven EsworthyHiu Chuen LokSteven OfferHomira BehbahaniSteven R. BrennerHong Sheng ChengSteven SilversteinHongbo GuStewart B. KirtonHongbo WangSubodh DubeyHongliang HeSubuhi KaulHongnan CaoSudhahar VaradarajanHongran YinSudip PaulHongtao JinSuhas Sampat KharatHongyan LiSujan K. MondalHon-Yi ShiSujeet KumarHoria PăunescuSuman AlishettyHortensia RodriguezSumet KongkiatpaiboonHosam O. ElansarySunbin DengHossam Saad El-BeltagiSung Noh HongHossein EslamiSung-Hyen LeeHoussem BoulebdSupandeep Singh HallanHoward A. YoungSuresh Ponnayyan SulochanaHrvoje BudinčevićSuresh VeeraperumalHsing I. HuangSusan A. FarrHsin-Yao WangSusana AmézquetaHsueh-Wei ChangSusana Anahi DandlenHuajun ZhengSusana Maria Henriques HenriquesHuang QiuSusanne KaeslerHui ZhaoSushil ChaudharyHui ZhuSushil Kumar ShakyawarHumberto Hernandez-SanchezSusumu OhyaHung-Chi ChengSuvik AssawHung-Pin TuSuzanne De La MonteHung-Yin LinSvend Borup JensenHye Suk LeeSvetlana KlementevaHyemin KimSvetlana KlinovaHyeone KimSwami Vetha Berwin SinghHyeong-Geug KimSwapna YellankiHyun Sung KimSwapnil Ganesh SanmukhHyun-Joon HaSwarnali RoyHyun-Ouk KimSwaroop Kumar PandeyIan JenkinsSyed A. A. RizviIan WymanSyed Ali Raza NaqviIbolya FülöpSyed ImamIbrahim H. EissaSyed Mohammed BasheeruddinIgor A. ZolotukhinSyed Najmul Hejaz AzmiIgor Borisowitsch BuchwalowSyed Nasir Abbas BukhariIgor JerkovićSyeda Abida EjazIlaria CampesiSyeda ShehzadiIlaria RubinoSylvain PeugetIleana CorvoSylvia Le DévédecIliyan IvanovSylvie AmuIlker S. BayerSzu-Cheng KuoIl-Kwon ParkSzymon JanczarIlze ŠtrumfaTabito KinoImari MimuraTadeusz KarczImtiaj HasanTadeusz KowalskiIndira ShrivastavaTae Hwan KimIndumathi SathisaranTaei MatsuiInês F. AntunesTae-il KimInes ManciniTaesup LeeIngrid CipakovaTahir FarooqIngrid Fricke-GalindoTakahiro KusukawaIngrid HerrTakanari NakanoInna MiroshnykTakane Kikuchi-UedaIoana CabaTakanori KanazawaIoannis ApostolopoulosTakashi YazawaIoannis KostasTakayuki FuruishiIoannis VamvakarisTakehiro KawashiriIogann TolbatovTakehisa HanawaIole VendittiTakeru MakiyamaIram MurtazaTakeshi SatoIrena Roterman-KoniecznaTakumi IshizukaIrene Gómez CruzTakuro NiidomeIrene SzijanTakuya TadaIreneusz KapustaTalib M. AlbyatiIrfan A. RatherTamaki EndohIrina M. Le-DeygenTamara ČačevIrina NegutTamás SoványIrina PopescuTamer SakrIrina TerekhovaTamsyn MontagnonIrina VelikyanTania PenchevaIrina ZarafuTanja J. MiličićIrshad SharafutdinovTanveer A. TabishIsaac Yves Lopes MacêdoTanyalak ParimonIsabel Legaz PérezTao YiIsabella RussoTao ZhangIsaiah ArkinTaoufiq BenaliIseli NantesTarek A. AhmedIslam HusainTaro ShimizuIsmael Martínez-CortésTatiana AndreaniIsmael Sánchez-GomarTatiana AntipovaIsmaheel O. LawalTatsuo Kandaİsmail ÖçsoyTatsuya MorimotoIsra AliTatsuya ShirahataIsrael AlshanskiTatyana P. KukinaIsrael Pérez-TorresTeemu Johannes MurtolaIstvan AntalTeerasak DamrongrungruangIstván KertészTeja Muralidhar KalidindiIstvan ZupkoTengfei SongItamar GonçalvesTeodora Alexa-StratulatIuliana AproduTeodorico C. RamalhoIuliana SpiridonTeodorico De Castro RamalhoIvan A. YaremenkoTeralı KeremIvan KosikTeresa SierraIvan S. KiselevTeruya KomatsuIvan SavicTeshamae S. MonteithIvan V. BogdanovTetsuro TagoIvana DelalleTetsuya KushikataIvana DjuricicThangavel PonrasuIvo PischelTheodore E. WarkentinIyan SopyanTheodoros EleftheriadisIzabela ChmielewskaTheodoros KarantanosIzabela FeckaTheodosia MainaIzabela JendrzejewskaTheoni KanellopoulouIzabela Sadowska-BartoszThilina U. JayawardenaJacek CapalaThomas E. PrisinzanoJacek KujawskiThomas J. J. MüllerJacek ŁyczkoThomas KlimkaitJacek NyczThomas KühneJacek OwczarekThomas MavromoustakosJacek PiatekThomas SciorJacek SzepietowskiThottethodi Subrahmanya Keshava PrasadJacek W. MorzyckiTianhui HuJacopo Junio Valerio BrancaTímea SzekerczésJacopo LucchettiTimofey LebedevJacopo SgrignaniTimothy G. BergerJacques ScheresTimothy SnowdenJae ChoTimur SalievJae Hyang LimTina Tičinović KurirJae Yeol LeeTiziano MarzoJaebong JangTiziano TuccinardiJaeseong OhTom GillJagdish JoshiTomas BlancoJaideep SandhuTomas G. VillaJaime Bustos-MartínezTomáš TobrmanJaime E. CharrisTomasz KubrakJaime EscalanteTomasz M. KarpińskiJaime PortillaTomasz RusakJaime Rubio-MartinezTomasz SawickiJakub MatusiakTomasz StompórJalal TaneeraTomica HrenarJames AngelastroTommaso GiovanniniJames B. FinkTomofumi YamamotoJames GruschusTonali Blanco-AyalaJames J. DriscollTor Henrik Anderson TvedtJames LettsToru IshikawaJán KozempelToru TaguchiJan L. MiljkovićToshihiko TashimaJan MirToshio TakahashiJan PetrToshitsugu FujitaJan SteenekampToyofumi SuzukiJan ZitkoTracy A. BrooksJana JaalTran Dang XuanJanakiram R. VangalaTravis T. DentonJane LiesveldTrina E. TalleiJanez IlašTsunehiko HiguchiJared Adolf-BryfogleTsun-Thai ChaiJari IntraTsvetelina VelikovaJaroslav PejchalTuğba GüngörJarosław ĆwikłaTugba Taskin TokJarosław J. PanekTuo MengJarosław PaluszczakTzer-Bin LinJaroslaw ZalewskiTzong-Shyuan LeeJason EriksenUbaldo ArmatoJason L. GuoUdaya S. TantryJason Tze Cheng TzenUjjal Kumar BhawalJavad AlizargarUlf Andersson ØromJavier Martinez-UserosUlrich SchubertJavier Menéndez-MenéndezUlrich WalterJavier Murciano-CallesUmberto MusazziJavier P. GisbertUmesh S. DeshmukhJavier VicarioUpendar Rao GollaJay AnandUrban BrenJay PenneyUrszula KarczmarczykJay StewartUsama Ramadan AbdelmohsenJayachandran VenkatesanVaibhav A. DixitJayalakshmi VallamkonduValdir Florencio Da Veiga JuniorJayapal Reddy MallareddyValentina BudaJazmin M. Pérez-RojasValentina GrossiJean Luc OlivierValentina StranieroJean Paul KamdemValentina UivarosiJean-Bosco JoudaValeria PistorioJean-Marc BarretValerie CarabettaJean-Philippe PelloisValério Monteiro-NetoJedlińska AleksandraVan Giau VoJeff WiluszVanessa BianconiJeffrey M. TingVanessa VieiraJeffrey Pradeep RajVanja TadićJeffrey SilberzweigVarun KumarJelena DinićVasil D. SimeonovJelena KnezevicVeera C. S. R ChittepuJelena PoljarevicVeera Ganesh YerraJelena Popović-DjordjevićVeijo HukkanenJelena RoganovicVelichka AndonovaJelena TošovićVendela ZetterqvistJelena VladićVenerando PistaràJeong-Hoon LimVera AlferovaJeongsoo YooVera DamuzzoJeong-tae LeeVera Marisa CostaJer-Yiing HoungVerica Aleksic SaboJerzy Jan JaroszewskiVeronica DoderoJerzy JuśkiewiczVeronika HuntosovaJeske WalterVesna JacevicJess VergisVesna Tumbas ŠaponjacJessica RosatiVesna V. PanićJesus Jimenez-BarberoVetriselvan SubramaniyanJesús Muñiz-SoriaVictor InfanteJhon Castaño-OsorioVictor M. Bolanos-GarciaJhy-Der ChenVíctor M. RiveraJia XiongVictoria GrishkoJiacan SuVictoria LeiroJiale YangVictoriya A. GeorgiyantsJia-Ling RuanViet-Khoa Tran-NguyenJialiu ZengVijay KotraJian YinVijay KumarJianfeng XiangVijay Kumar SiripuramJiann-Ruey HongVijay MishraJichun ChenVijayaprakash SuppiahJie SunVikas Anand SaharanJi-Kan RyuVikas KumarJimin GuoVikram PrasadJin ChenViktor ZvarychJin WangViliana Eduardova GuglevaJinbo FeiVilma KaškonienėJing LiuVinayak KhodadeJing-hua YangVincenzo QuagliarielloJinming HanVincenzo SorrentiJinn-Rung KuoVineeth Chandran SujaJinwei ZhangViola CalabròJin-Yue SunVioleta PurcarJirapornchai SuksaereeVipan KumarJitlada VichapongViswas Raja SolomonJiun-Wen GuoVisweswara Rao PasupuletiJo CaersVita DolžanJoachim ClosVitaliy GarginJoana A. LoureiroVittoria ColottaJoana BickerVittoria Di MauroJoana M. GasparVittorio MazzarelloJoana Silva RibeiroVivek TrivediJoanna Dagmara FedorowiczVlad Constantin TofanJoanna GiebułtowiczVlad PădureanuJoanna JaworskaVlad PorumbJoanna ListosVladiana TuriJoanna MatysiakVladimir ChubanovJoanna MikloszVladimir D. AjdačićJoanna SobiakVladimir DyakonovJoanna SuliburskaVladimir I. KalininJoão Azevedo-SilvaVladimir Igorevich MakarovJoão Gaspar MarquesVladimir N. SilnikovJoão Henrique Ghilardi LagoVladimir P. BerishviliJoaquim CarrerasVladimir V. ZarubaevJoaquín María CamposVladimir VimbergJochen BodemVrba JiriJoe M. ViljoenWael HegazyJoeri Jan PenWajid ZamanJohanan Christian PrasanaWaldemar GoldemanJohannes FruehWaldemar TurskiJohannes LützenkirchenWaleed Bakry SuleimanJohn DzimianskiWalied AbdoJohn G. HardyWalter MurilloJohn H. MillerWang FangJohn HortonWangsa Tirta IsmayaJohn T. BatesWanpen ChaicumpaJoias HammanWasan KatipJolanta Orzelska-GórkaWaseem AhmedJolanta PolakWaseem El-HuneidiJonathan RoydsWashim KhanJongsun ParkWasuke MoriJonnathan Guadalupe Santillán-BenítezWataru HakamataJoo Hyun OWayne CarterJoong ShimWei Guang SeetohJordi TeixidóWei XuJörg BreitkreutzWeichao ChangJorge A. GuimarãesWei-En YuanJorge CervantesWeifeng LinJorge GonçalvesWeijian BeiJorge H. LeitãoWeiwei HanJorge TrillerasWen-Bin YangJos L. EerselsWen-Chin LeeJos PaulusseWendy M. WalwynJose Alegre-MartínWenhua ZhengJosé Antonio RodríguezWenjie WangJosé Carlos Tavares CarvalhoWenjun (Chris) ZhangJosé Ernesto BelizarioWenquan YuJosé Fernando Oñate-GarzónWen-Tsong HsiehJosé JusticiaWerner MüllerJosé L. FusterWieslawa Agnieszka FogelJose Manuel LopesWilliam A. DennyJosé Miguel Seguí-RipollWilliam FolkJosé Pedraza ChaverriWilliam N. SetzerJosé Pedro Cerón-CarrascoWilliam SchwanJosé Rafael De AlmeidaWim BuijsJosé X. SoaresWing-Leung WongJosef FinstererWiramon RungratanawanichJosefa LeónWissem MnifJoseph A. DiVerdiWładysław P. WęglarzJoseph FalkinhamWojciech KalasJoseph Thomas OrtegaWojciech KochJosipa VlainićWojciech PiekoszewskiJovanny GomezWolfgang FischerJuan AyalaWolfgang WadsakJuan Carlos GrignolaWolfram S. KunzJuan CastilloWoong Sub ByunJuan David Ospina-VillaWubshet TesfayeJuan F. GonzálezWufu ZhuJuan José Ramos-RodríguezXavier BrazzolottoJuan José Torrado DuránXiaofei LiJuan Manuel Germán-AcacioXiaojie XuJuan Pablo de Rivero VaccariXiaojun WeiJuan Pedro Martínez-RamónXiao-Kun OuyangJuan PellicoXiaoliang QiJuei-tang ChengXiaoping PuJulia Christina GrossXiaopo ZhangJulia Matzenbacher Dos SantosXiaoyu HaoJuliana LunaXiaoyuan ChenJuliano GaravagliaXiaxia DiJulie TuckerXin ChenJulien DiharceXin SunJulien PetrignetXin ZhangJulien Saint-PolXinda LiJulien TailhadesXingang YaoJuliette HadchouelXingshun QiJulio C. Rivera-LeyvaXuelin ZhouJulita KulbackaXuping LiJun MaXu-Qiao ChenJun ToyoharaXuyong LiJunbo GongYa LiJunbo ZhangYaacov Ben-DavidJung SunwooYa-Hsuan ChangJunghyun KimYajing WangJungmoo HuhYan V. ZubavichusJun-Goo JeeYan XuJun-ichi SawadaYana FeodorovaJun-Jen LiuYang HuJunzi WuYang LiuJu-Ock NamYan-Ming XuJurga BūdienėYann SeimbilleJürgen MartensYanping YuJuri TimonenYao WangJu-Seop KangYaroslav A. AndreevJustas DapkūnasYaser Daanial KhanJustus G. GarwegYash GuptaJustyna Knapik-KowalczukYasser A. El-AmierJustyna ŻwawiakYasuhito IshigakiJwu-Lai YehYasumichi InoueJyh-Fei LiaoYasunari KandaJyoti RanaYasunari MatsuzakaKadir OzaltinYasunori YaoitaKai ZhangYasutaka KakinokiKai-Wen ChengYasuyuki TokinagaKai-Wen HsuYesim Gokmen-PolarKak Shan ShiaYesu AddepalliKálmán ImreYeu SuKamal MandalYi ZouKamal ShahYihenew Simegniew BirhanKamalendra SinghYing WangKameliya AnichinaYinghuai ZhuKamenna VutovaYingkun QiuKamil KuderYishen ZhuKamol DeyYi-Ting LinKampanart HuanbuttaYlenia SpissuKanchan KohliYoana Dimitrova Kiselova-KanevaKandhasamy SowndhararajanYohan N'GuyenKanwal RehmanYohei ShirakamiKarel Smetana Jr.Yoichi ShimizuKaren Ildico Hirsch-ErnstYong WangKarien LabuschagneYong XuKarima DjabaliYong-Ai XiongKarol P. NartowskiYongfeng GaoKarol WróblewskiYonghong ZhuKarola Vorauer-UhlYongming BaoKarolina Chilicka-HebelYosef ShamayKarolina SłoczyńskaYoshiaki KitamuraKarolina Szewczyk-GolecYoshihiko NakataniKároly GulyaYoshihiro NoguchiKatalin KristóYoshihiro ShidojiKatanasaka YasufumiYoshihisa UenoyamaKatarína ValachováYoshikane YamauchiKatarina VukojevićYoshikazu KitanoKatarzyna BączekYoshikazu UchidaKatarzyna Bialik-WąsYoshiko MiuraKatarzyna Bober-MajnuszYoshinori InagakiKatarzyna IżykowskaYoshitaka ShimotaiKatarzyna JelonekYosra A. HelmyKatarzyna Kiec-KononowiczYotsanan WeerapolKatarzyna Kosicka-NoworzyńYouness El BakriKatarzyna MichalskaYouness LimamiKatarzyna SkrypnikYounghoon JangKatarzyna SobierajskaYoung-IL JeongKatarzyna Sykłowska-BaranekYoung-Kwon SeoKatarzyna Winsz-SzczotkaYu CaiKatarzyna Wolny-KoładkaYu SunKatharigatta Narayanaswamy VenugopalaYu TakanoKatharina BeyerYuan-Bin ChengKatharina RumpYuda ChenKatherine GillYuefei JinKathleen ClaesYuemin BianKathrin Bellmann-SickertYue-Zhou ZhangKathryn Y. BurgeYuhki SatoKatia IskandarYu-Hsiang KuanKatia StefanantoniYu-Hua ChaoKatona GáborYuji ShimizuKatsuhiko KatoYukihiko MomiyamaKatsunori YoshidaYuko MorikawaKatyayani TatipartiYulia E. GorshkovaKavindra K. KesariYulia V. NelyubinaKavita SharmaYulin RenKay-Dietrich WagnerYu-Lung HsuKazem NazariYun ShiKazimierz KuliczkowskiYunfeng ZhaoKazuhito AsanoYunlong ShiKazutomo SuzueYunusa UmarKazuya MaedaYun-Wen ZhengKe DingYupei LiKe MaYu-Shun YangKedong SongYusof KamisahKee-Lung ChangYuting YuanKen UrishYuto UedaKen-Ichiro MatsumotoYu-Wei ChangKevin CoulouresYuya TakakuboKevin ItaYves DelnesteKeyvin DarneyYves-Jacques SchneiderKhac-Minh ThaiZahari VinarovKhajornsak TragoolpuaZalba SaraKhaled M. DarwishŽarko MitićKhalid Mohamed El-SayZarrin BasharatKhan BushraZavala-Rivera PaulKhawla S. KhashanZbârcea Cristina ElenaKhurshid AhmadZbigniew J. WitczakKi Soo ParkZbigniew Jan LeśnikowskiKi Su KimZdenék KejíkKiattawee ChoowongkomonZeenat MirzaKichul ShinZeliha SelamoğluKieffer CharlineZeljka KoradeKiessoun KonatéZeljka VanicKi-Jung PaengZhaoxi SunKimberly PerezZhe LiuKinga Kowalska-DuplagaZhen ChengKinga MolnárZhenbo HanKiran SandhuZhi ShiKiran TotiZhiguo ZhangKirill TkachenkoZhihao LiuKirsten R. Müller-VahlZhihong XuKishor MazumderZhijie WangKishwor PoudelZhipeng HuKit Leong CheongZhipeng TaoKiyomitsu NemotoZhiqiang KuKiyoshi TakagiZhirong LiuKiyotaka UchiyamaZhong-Ru XieKi-Young KimZhuoxun WuKlaudia Izabela KocurekZihao OuKlaus-Dieter SchlüterZijian ZhangKollarigowda RavichandranZijing LiKollur Shiva PrasadZili SideratouKonrad A. SzychowskiZilong WangKonstantin V. KudryavtsevZixin ChenKoraljka Gall-TrošeljZizy Ibrahim ElbialyKorbtham SathirakulZlatko KopeckiKormos ViktóriaZofia MazerskaKosta PopovićZograb MakiyanKouhei YamadaZoltán BaráthKrishan KumarZoltán PethőKrishna K. SharmaZoltán SzűcsKrishna PatelZoltán ÚjhelyiKrishnan ThirumoorthyZoubir DjeradaKrisztian GasparZrinka RajićKrisztina LudányiZsolt GállKrzesimir CiuraZulfan ZazuliKrzysztof SztankeZuzanna RzepkaKuan-Han LeeZygmunt Warzecha


